# Single-cell RNA sequencing reveals a high-resolution cell atlas of petals in *Prunus mume* at different flowering development stages

**DOI:** 10.1093/hr/uhae189

**Published:** 2024-07-10

**Authors:** Yuhong Guo, Xiling Chen, Jinhong Li, Qi Wang, Shuangyu Zhang, Nuoxuan Liu, Yanlong Zhang, Tengxun Zhang

**Affiliations:** College of Landscape Architecture and Arts, Northwest A&F University, Yangling, Shaanxi 712100, China; College of Landscape Architecture and Arts, Northwest A&F University, Yangling, Shaanxi 712100, China; College of Landscape Architecture and Arts, Northwest A&F University, Yangling, Shaanxi 712100, China; College of Landscape Architecture and Arts, Northwest A&F University, Yangling, Shaanxi 712100, China; College of Landscape Architecture and Arts, Northwest A&F University, Yangling, Shaanxi 712100, China; College of Landscape Architecture and Arts, Northwest A&F University, Yangling, Shaanxi 712100, China; College of Landscape Architecture and Arts, Northwest A&F University, Yangling, Shaanxi 712100, China; College of Landscape Architecture and Arts, Northwest A&F University, Yangling, Shaanxi 712100, China

## Abstract

*Prunus mume* (mei), a traditional ornamental plant in China, is renowned for its fragrant flowers, primarily emitted by its petals. However, the cell types of mei petals and where floral volatile synthesis occurs are rarely reported. The study used single-cell RNA sequencing to characterize the gene expression landscape in petals of *P. mume* ‘Fenhong Zhusha’ at budding stage (BS) and full-blooming stage (FS). Six major cell types of petals were identified: epidermal cells (ECs), parenchyma cells (PCs), xylem parenchyma cells, phloem parenchyma cells, xylem vessels and fibers, and sieve elements and companion cells complex. Cell-specific marker genes in each cell type were provided. Floral volatiles from mei petals were measured at four flowering development stages, and their emissions increased from BS to FS, and decreased at the withering stage. Fifty-eight differentially expressed genes (DEGs) in benzenoid/phenylpropanoid pathway were screened using bulk RNA-seq data. Twenty-eight DEGs expression increased from BS to FS, indicating that they might play roles in floral volatile synthesis in *P. mume*, among which *PmBAHD3* would participate in benzyl acetate synthesis. ScRNA-seq data showed that 27 DEGs mentioned above were expressed variously in different cell types. *In situ* hybridization confirmed that *PmPAL2*, *PmCAD1*, *PmBAHD3*,*5*, and *PmEGS1* involved in floral volatile synthesis in mei petals are mainly expressed in EC, PC, and most vascular tissues, consistent with scRNA-seq data. The result indicates that benzyl acetate and eugenol, the characteristic volatiles in mei, are mostly synthesized in these cell types. The first petal single-cell atlas was constructed, offering new insights into the molecular mechanism of floral volatile synthesis.

## Introduction

Floral fragrance is an important trait of ornamental plants that are used in perfume production and plays a crucial role in attracting pollinators and adapting to biotic and abiotic stresses. Floral volatiles are a mixture of secondary metabolites in plants, which are divided into three major classes: terpenoids, benzenoids/phenylpropanoids (BPs), and fatty acid derivatives [1]. These volatiles are mainly synthesized in petals [2]. Some genes involved in floral volatile synthesis exhibit specific or high expression in epidermal cells (ECs) of petals, such as linalool synthase gene (*CbLIS*) in *Clarkia breweri*, benzoic acid methyltransferase gene (*AmBAMT*) in *Antirrhinum majus*, and orcinol *O*-methyltransferase gene (*RhOOMT*) in rose petals [3–5]. Several structural genes are involved in the biosynthesis pathway of a given floral volatile [6]. However, whether these genes are expressed in a single cell remains to be proven.

*Prunus mume* (mei), a traditional ornamental flower in China, has a long cultivation history. It belongs to the *Prunus* genus of Rosaceae family and originates in the Yangtze River basin in southern China, which usually blooms in early spring. Furthermore, mei produces a characteristic floral fragrance compared to other *Prunus* species, such as peach and apricot [7]. Thus, it is important to explore the potential molecular mechanism of floral volatile synthesis in mei to develop aromatic cultivars in the *Prunus* genus. Some studies have shown that benzaldehyde, benzyl alcohol, benzyl acetate, and eugenol are characteristic aroma components of mei [8]. These volatiles are synthesized through the BP pathway [9]. A series of genes involved in floral scent biosynthesis in mei have been studied using bulk RNA-seq: phenylalanine ammonia-lyase gene (*PmPAL*), 4-coumarate-CoA ligase gene (*Pm4CL*), cinnamate-CoA ligase gene (*PmCNL*), cinnamate 4-hydroxylase gene (*PmC4H*), cinnamoyl-CoA reductase gene (*PmCCR*), cinnamyl alcohol dehydrogenase gene (*PmCAD*), benzyl alcohol acetyltransferase gene (*PmBEAT*), and eugenol synthase gene (*PmEGS*) [10–12]. However, such studies have mostly selected the whole flower as the experimental material. Thus, it might be difficult to explain the expression pattern of key genes in different cell populations. Assuming that scent-producing cells can be separated from the rest of the cells in the entire petal, the priority of key genes in floral volatile synthesis will be better explored [13].

Single-cell RNA sequencing (scRNA-seq) is a new technology that analyses transcriptomes of thousands of single cells simultaneously, identifies unknown cell types, and explores the potential roles of different cell types in plant organs to highlight functional specialization in many plants, such as *Oryza sativa* roots, poplar stem, and strawberry leaves [14–17]. ScRNA-seq has also been applied to investigate the cell types of petals and floral fragrance synthesis at the cellular level. For example, cell types in *Nicotiana attenuata* corolla were identified using scRNA-seq, and the molecular mechanism of the cell type-specific metabolism of benzyl acetone biosynthesis was revealed [13]. However, there are no relevant studies on non-model plant petals at the single-cell level. Analysis of a single-cell transcriptome in mei petals will enable us to identify the major cell types, focus on the expression dynamics of differentially expressed genes (DEGs) among different cell clusters, and discover cellular interaction and diversity at the precise time of volatile synthesis. It is also a reference for further understanding the molecular mechanism underlying the cell type specificity of BP biosynthesis.

**Figure 1:**
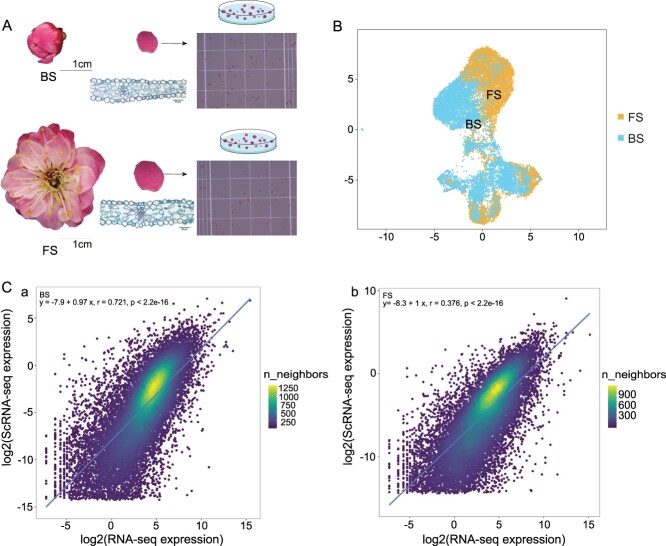
Isolation and analysis of single-cell transcriptomes from mei petals. **A** Petal protoplast extraction at BS and FS. **B** UMAP visualization of samples at BS and FS. **C** Correlation analysis of scRNA-seq profiling and bulk RNA-seq at BS (a) and FS (b), respectively.

In this study, *P. mume* ‘Fenhong Zhusha’ (FHZS) petals at budding stage (BS) and full-blooming stage (FS) were selected as the main materials to construct a single-cell gene expression map of woody plant petals using scRNA-seq. Floral volatiles and bulk RNA-seq data of variety ‘FHZS’ petals at four flowering development stages [BS, initial flowering stage (IFS), FS, and withering stage (WS)] were measured. Through combining scRNA-seq and bulk RNA-seq transcriptome data, candidate genes related to floral volatile synthesis in mei petals were screened, and their expression patterns in different cell types were analysed at BS and FS. The main expression sites of genes involved in floral volatile biosynthesis in mei petals were verified at the cellular level via *in situ* hybridization, enhancing the systemic understanding of the mechanism of floral volatile synthesis.

## Results

### Single-cell transcriptome sequencing of *P. mume* petals

ScRNA-seq on the petals of variety ‘FHZS’ was performed using the 10X Genomics Chromium platform (Fig. 1A). Free protoplasts from petals at BS and FS were obtained by enzyme digestion. After cell viability testing, 20 000 cells of each sample were used to construct two libraries sequenced by the PE150 mode of the Illumina sequencing platform. In total, 7983 and 8965 high-quality cells were successfully captured from petals at BS and FS, respectively. Correspondingly, 478 182 852 and 449 158 706 reads were obtained, with 97.2% and 97.8% valid barcodes, respectively, and 91.0% and 92.1% of reads were confidently mapped to *P. mume* var. *tortuosa* genome. A total of 19 072 and 18 296 genes were identified through scRNA-seq of petals at BS and FS, with 3358 and 2646 median genes per cell. The median unique molecular identifier (UMI) counts per cell were 11 973 and 9390, respectively (Table S1, see online supplementary material). Cells from two flowering development stages were visualized on a uniform manifold approximation and projection (UMAP) plot (Fig. 1B). At the same time, bulk RNA-seq of petals was performed at four flowering development stages. The datasets are shown in Tables S2 and S3 (see online supplementary material). Pearson correlation analysis was used to compare gene expression between scRNA-seq and bulk RNA-seq profiles. Results showed strong correlations (R = 0.736 and 0.721 for petals at BS and FS, respectively; *P* < 2.2e-16; Fig. 1C).

### Identification of cell types in *P. mume* petals

**Figure 2:**
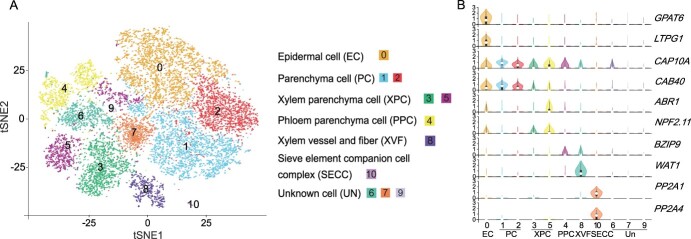
Cell cluster identification of single-cell transcriptomes from mei petals. **A** Visualization of the 11 cell clusters. **B** Violin plots show the homologous gene expression distribution of the reported marker genes in different cell types in mei.

After filtering out low-quality cells, an unsupervised clustering analysis of 16 948 cells was conducted. Eleven clusters were formed (Fig. 2A). Because there were no cell-specific marker genes and a high-revolution atlas in petals reported, clusters were assigned based on orthologs of single-cell markers in other plants. Cluster 0 was ECs, which contained the marker genes *GPAT6* and *LTPG1* that are known to be involved in the export of epidermal components and cutin biosynthesis in petal formation [18, 19]. Clusters 1 and 2 were parenchyma cells (PCs) for high expression of *CAP10A* and *CAB40* (orthologs of *FvLHCB2.1*), which are marker genes for PC [17, 20]. Clusters 3 and 5 were xylem parenchyma cells (XPCs) and expressed the marker genes *NPF2.11* and *ABR1* [21, 22]. Cluster 4 was defined as phloem parenchyma cells (PPCs) because of the expression of *bZIP9* that was specifically expressed in PPCs [23]. It was speculated that cluster 6 might be likely PPCs due to the enrichment of the same genes with cluster 4. Cluster 8 was xylem vessels and fibers (XVFs) with the marker gene *WAT1*, which determines the secondary cell wall thickness of wood fibers and is associated with developing XVFs in *Arabidopsis thaliana* [16, 24]. *PmWAT1*, expressed predominantly in xylem vessels, was observed using *in situ* hybridization (Fig. S1, see online supplementary material). Cluster 10 was the sieve element companion cell complex (SECC) in phloem and contained the marker genes *PP2-A1* and *PP2-A4* [25, 26]. Cell types of clusters 6, 7, and 9 could not be determined using the current marker genes and were named unknown cells (Fig. 2B).

These cell-specific DEGs were enriched in various biological processes in Gene Ontology terms (Fig. S2a, see online supplementary material). DEGs in cluster 0 were rich in lipid metabolic process and fatty acid biosynthetic process, which were related to ECs. DEGs in clusters 1 and 2 were enriched in ribosome biogenesis and organonitrogen compound metabolic process. Clusters 3 to 5, 8, and 10 were thought to be vascular tissues, and DEGs in these clusters were mainly involved in signal molecule conduction and transport. DEGs in clusters 6, 7, and 9 were unavailable for further analysis. Based on the Kyoto Encyclopedia of Genes and Genomes annotation, the enriched pathways in ECs (cluster 0) were mostly involved in metabolic pathways, fatty acid metabolism, steroid biosynthesis, amino sugar and nucleotide sugar metabolism, and so on. DEGs in PCs (clusters 1 and 2) were primarily related to photosynthesis-antenna proteins of petals. DEGs in vascular tissues (clusters 3 to 5, 8, and 10) were mainly enriched in ribosome, secondary metabolic biosynthesis, endocytosis, and plant hormone signal transduction pathways (Fig. S2b, see online supplementary material).

### Discovery of cell-specific marker genes in *P. mume* petals

**Figure 3:**
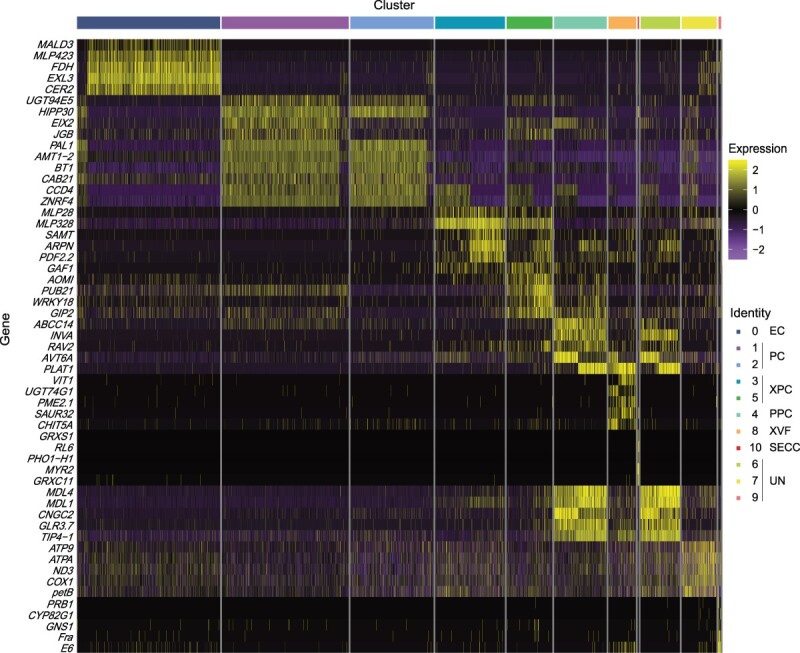
Expression heatmap of cell-specific marker genes in 11 clusters of petals. The expression value of each gene was z-score normalized. EC, epidermal cell; PC, parenchyma cell; PPC, phloem parenchyma cell; SECC, sieve element companion cell complex; UN, unknown cell types; XPC, xylem parenchyma cell; XVF, xylem vessel and fiber.

After identifying cell clusters, the number of relevant upregulated DEGs ranged from 447 in cluster 10 to 1834 in cluster 0 (Fig. S3, see online supplementary material). The top five cluster-specific genes in each cluster were collected (Fig. 3). *MALD3*, *MLP423*, *FDH*, *EXL3*, and *CER2* were highly enriched in ECs (cluster 0). *MALD3* and *FDH* (the orthologous of *NaKCS10*), were the epidermal-specific marker genes in *A. thaliana* and *N. attenuata*, respectively [13, 19]. *AtMALD3* was found to be responsible for trafficking cutin and wax to the plant surface for assembly and deposition of the cuticle, and *NaKCS10* was involved in cuticular wax and cutin biosynthesis. *PaEXL3* in *Prunus armeniaca* might be associated with lipid metabolism to protect plants against cold stress [27]. *UGT94E5*, *JGB*, *EIX2*, *CAB21*, and *AMT1–2* were highly expressed in PCs (clusters 1 and 2). CAB (chlorophyll *a*/*b* binding protein of LHCII type) proteins are related to photosynthesis, providing energy for plant development [28]. *PmAMT1–2* might also be related to floral volatile synthesis because *PmAMT1–2* expression was positively correlated with benzyl acetate and benzaldehyde production in *P. mume* [10]*.* The expression of *MLP328*, *GAF1*, *AOMI*, *PUB21*, and *WRKY18* showed high specificity in XPCs (clusters 3 and 5). *ABCC14, INVA, RAV2, AVT6A,* and *PLAT1* were expressed more in PPCs (cluster 4). *VIT1*, *UGT74G1*, *PME2.1*, *SAUR32*, and *CHIT5A* were mainly expressed in cluster 8 and considered as the marker genes in XVFs. *PmSAUR32* was highly expressed in *P. mume* ‘WuYuyu’ stems, which was involved in hormone and sugar metabolism [29]. *MYR2*, *CLE25*, *GRXS1*, *RL6*, and *PHO1-H1* were mostly expressed in cluster 10, which were thought to be the marker genes in SECC (Table S4, see online supplementary material). *AtPHO1-H1* was expressed in the vascular cylinder of roots and shoots in *A. thaliana* under phosphate-deficient conditions to support plant growth and survival [30]. These marker genes would contribute to recognizing the petal cell types of mei.

### Dynamic changes in floral volatiles and potential genes related to their synthesis in *P. mume* petals

**Figure 4:**
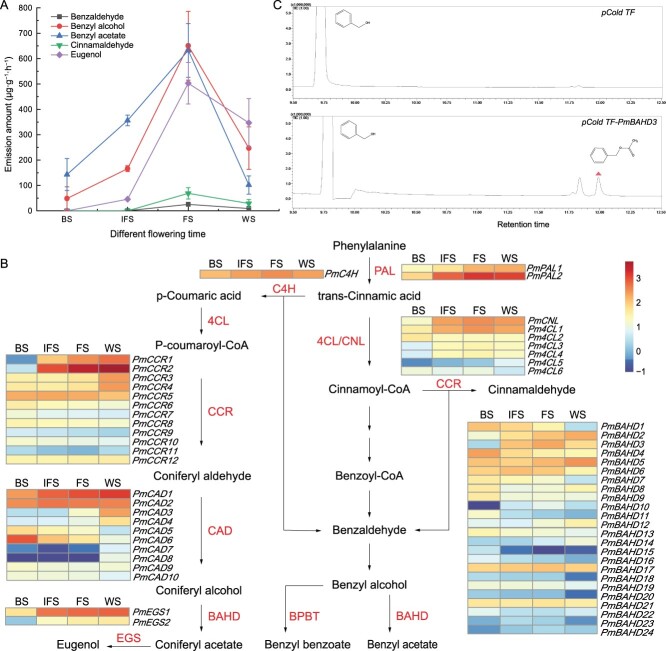
Correlation analysis of floral volatile compounds emission and the related regulated genes in biosynthesis. **A** Dynamic changes in the main floral volatiles released at four flowering development stages of mei. **B** Heat map of transcriptome expression of 58 structural genes related to floral volatile biosynthesis from BS to WS. FPKM values were log10 transformed. BS, budding stage; FS, full-blooming stage; IFS, initial flowering stage; WS, withering stage. **C**  *In vitro* analysis of the enzyme activity of PmBAHD3.

The floral volatiles of variety ‘FHZS’ petals were analysed at four flowering development stages using gas chromatography–mass spectrometry (GC–MS). Benzyl alcohol, benzyl acetate, eugenol, cinnamaldehyde, and benzaldehyde, which originated from BP biosynthesis, were detected. Their contents during release increased from BS to FS and decreased at WS, reaching the peak at FS. The amounts of benzaldehyde and cinnamaldehyde were low at the four stages (Fig. 4A).

Fifty-eight DEGs involved in BP pathway were screened using bulk RNA-seq transcriptome data of petals (Table S5, see online supplementary material): 2 *PmPALs*, 1 *PmC4H*, 1 *PmCNL*, 6 *Pm4CLs*, 10 *PmCADs*, 12 *PmCCRs*, 24 *PmBAHDs*, and 2 *PmEGSs* (Fig. 4B). The expression of *PmPAL2*, *PmC4H*, *PmCNL*, *Pm4CL1,3,4*, *PmCCR5*, and *PmBAHD3,6,10,17,24* increased from BS to FS and decreased at WS. *PmCAD2* expression increased from BS to IFS and decreased slightly from IFS to WS. Moreover, the expression of *PmPAL1*, *PmCAD1,3,4*, *PmCCR1,2,3,4*, *PmBAHD2,5,12,14*, and *PmEGS1,2* continued to increase from BS to WS, whereas that of *PmCCR4* and *PmBAHD5,12,14* decreased slightly from BS to IFS. *PmBAHD8* expression significantly decreased from BS to IFS and increased slightly from IFS to WS. Overall, the expression patterns of 28 structural genes increased from BS to FS, in accordance with the emission of the floral scent, indicating that they might play vital roles in floral volatile synthesis in *P. mume*.

The floral fragrance profiles in mei were dominated by benzyl acetate and eugenol. It has been reported that PmBAHD5 (orthologs of PmBEAT36) catalyzed benzyl alcohol to benzyl acetate and *PmBAHD3* (the homologous gene of *PmCFAT1*) was involved in eugenol synthesis [7, 31]. Because of the substrate diversity of acyltransferases, this study delved deeper into the functional roles of *PmBAHD3* by heterologous expression in *Escherichia coli*. The purified recombinant protein was obtained (Fig. S4, see online supplementary material), and enzymatic activity was performed *in vitro* with benzyl alcohol as a substrate. Benzyl acetate was detected in the reaction production using GC–MS (Fig. 4C). PmBAHD3 would function on benzyl acetate synthesis, suggesting that benzyl acetate formation in *P. mume* might be the result of a combination of multiple alcohol acyltransferases.

### Exploration of cell types related to floral fragrance synthesis in *P. mume* petals

We analysed the cells percentage of ‘FHZS’ petals at different flowering development stages. From BS to FS, the proportions of ECs decreased sharply, whereas that of PCs increased obviously. The percentages of vascular tissue cells changed slightly between BS and FS (Fig. S5a, see online supplementary material). DEG expression in each cell type of ‘FHZS’ petals at BS and FS was also obtained, which provided data to further analyse the expression of genes involved in floral fragrance synthesis in different cell types. In general, there were 2169 upregulated and 3132 downregulated genes at FS compared with those at BS (Fig. S5b, see online supplementary material).

**Figure 5:**
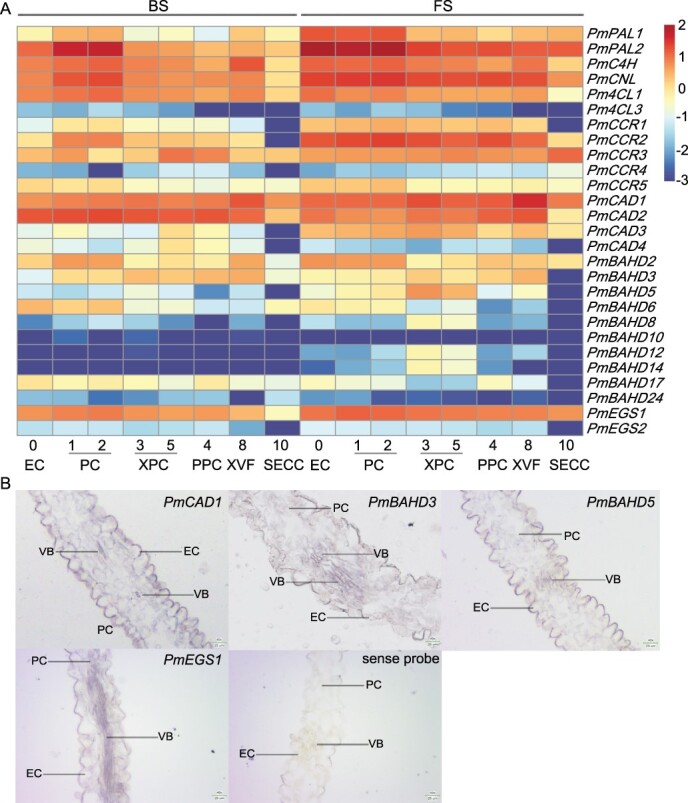
Cell-specific distribution of genes related to floral fragrance biosynthesis. **A** Heat map of the expression of 27 genes related to floral volatile synthesis in a single-cell transcriptome. Expression values were log10 transformed. BS, budding stage; FS, full-blooming stage. **B** Localization of the expression of four genes involved in benzyl acetate and eugenol synthesis in ‘FHZS’ petals by *in situ* hybridization. Bar = 25 μm. EC, epidermal cell; PC, parenchyma cell; VB, vascular bundle.

To further understand the cell types related to floral fragrance synthesis, this study explored the expression patterns of the 28 candidate genes mentioned above in scRNA-seq data: 2 *PmPALs*, 1 *PmC4H*, 1 *PmCNL*, 3 *Pm4CLs*, 5 *PmCCRs*, 4 *PmCADs*, 10 *PmBAHDs*, and 2 *PmEGSs*. Among them, 27 genes were detected, except for *Pm4CL4* (Table S6, see online supplementary material). The expression of 10 genes (*Pm4CL3*, *PmCCR4*, *PmCAD4*, *PmBAHD8,10,12,14,17,24*, and *PmEGS2*) was low in each cell type at BS and FS. The expression of 13 genes, including *PmPAL1,2*, *PmC4H*, *PmCNL*, *PmCCR1,2,3,5*, *PmCAD1,3*, *PmBAHD2,5*, and *PmEGS1* was higher at FS than at BS in all cells we obtained. Although these genes are expressed in almost every cell type, their expression varies among different cell types. For instance, the expression levels of *PmPAL1,2*, *PmC4H*, *PmCNL*, and *PmCCR5* in ECs and PCs were higher than those in other cell types at FS. The expression levels of *PmCCR1* and *PmCCR2* were slightly higher in PCs at BS and FS, whereas that of *PmCCR3* was higher in SECC. The expression of *PmCAD1* and *PmCAD3* was higher in XPCs and XVFs. *PmBAHD2* was expressed mainly in ECs and PPCs, which increased markedly from BS to FS. Despite its significant increase from BS to FS, *PmBAHD5* expression in PCs and XPCs was higher than that in other cell types. *PmEGS1* expression was slightly higher in PCs at FS. Besides, the expression levels of *PmCAD2* and *PmBAHD3,6* were lower in all cells at FS than in those at BS. *PmCAD2* expression was slightly higher in ECs, XPCs, PPCs, and XVFs at FS. *PmBAHD3* was also expressed in each cell type at FS, except for SECC, and exhibited higher expression in XPCs, PPCs and XVFs at FS. However, *PmBAHD3* expression increased significantly in ECs from BS to FS. There were no significant differences in *Pm4CL1* expression at these two flowering development stages, but its expression in ECs significantly increased from BS to FS (Fig. 5A).

Furthermore, the main expression sites of several genes, including *PmPAL2*, *PmCAD1*, *PmEGS1*, *PmBAHD3*, and *PmBAHD5*, were confirmed by *in situ* hybridization. As shown in Fig. S6 (see online supplementary material), *PmPAL2* expression in all cell types at FS was higher than that in cells at BS. And *PmPAL2* was primarily expressed in PCs at BS, whereas its expression in ECs and PCs was higher at FS, suggesting that scRNA-seq data were reliable. The cell types where the other four genes were expressed at FS were detected. *PmCAD1* and *PmEGS1* were expressed in each cell type of petals. Although *PmBAHD3* and *PmBAHD5* were detected in most cells, *PmBAHD3* expression in PPCs and XVFs was higher than that in others and *PmBAHD5* was highly expressed in XPCs, consistent with the scRNA-seq data. These results suggested that floral volatiles might be synthesized in ECs, PCs, and most vascular tissue cells of *P. mume* petals (Fig. 5B). These results also indicated the reliability of cell cluster classification in this study (Fig. 2).

## Discussion

*P. mume* is famous for its unique fragrance, which mainly comes from its petals [11]. Petals are generally composed of ECs, PCs, and vascular tissues [32]. Previous studies showed that PCs or ECs of petals is involved in volatile synthesis [33, 34]. Indeed, studies on *A. majus* showed that petal adaxial epidermal cell is the main site to produce floral fragrance [4]*.* Subepidermal parenchyma cells of ray florets of *Centaurea cyanus* exhibit many features of scent-producing cells*,* confirming their involvement in the production of volatile aromatic compounds in petals [35]. However, the specific cell types or layers in mei petals where floral volatiles generally occur remain unclear. ScRNA-seq has made it possible to annotate cell types of petals, which is a crucial step for subsequent analysis. A previous study discovered the major cell types in *N. attenuata* corolla by scRNA-seq, including ECs, PCs, phloem, xylem, and chlorenchyma cells, and benzyl acetone was mainly synthesized in ECs [13]. In this study, ECs, PCs, and vascular bundles were observed in ‘FHZS’ petals (Fig. S7, see online supplementary material), and six major cell types were identified using scRNA-seq (Fig. 2A). The cell-specific marker genes previously identified were validated in *P. mume*, including *PmWAT1* (Fig. S1, see online supplementary material), and novel marker genes of *P. mume* petals were provided. For instance, *PmMALD3* and *PmFDH* were specifically expressed in ECs, *PmCAB21* and *PmAMT1–2* were highly expressed in PCs, and *PmSAUR32* was mostly expressed in vascular tissues (Fig. 3). These novel marker genes could be used to identify the cell types of woody plant petals.

The emission of floral scent compounds exhibits temporal rhythms [6]. In ‘FHZS’ petals, the emission levels of unique volatile compounds originating from the BP pathway, such as benzyl alcohol, benzyl acetate, and eugenol, increased sharply from BS to FS and decreased at WS (Fig. 4A), consistent with previous studies [8, 36]. A series of structural genes are involved in the biosynthesis of benzyl acetate and eugenol, which are important characteristic floral scent compounds in mei [10]. PAL is the first and key enzyme in BP pathway that catalyzes the conversion of *trans*-cinnamic acid from Phe [6]. Then, *trans*-cinnamic acid generates eugenol and benzyl acetate via the C_6_–C_3_ and C_6_–C_1_ pathways, respectively, with the involvement of enzyme genes such as *C4H*, *CNL*, and *4CL* [6]. The expression levels of *PmPAL2*, *PmC4H*, *PmCNL*, and *Pm4CL1* were consistent with the emission levels of benzyl acetate and eugenol from BS to WS, suggesting that they may participate in their synthesis. *PmCCR1*,*2*,*3* and *PmCAD1* expression continued to increase from BS to WS and showed a strong correlation with the emission level of eugenol. PmCCR1,2,3 might provide coniferyl aldehyde, which was converted into coniferyl alcohols by PmCAD1 for eugenol formation. PmBAHD3 (ortholog of PmCFAT1) played a role in eugenol synthesis and might catalyze coniferyl alcohol to coniferyl acetate [31] that was the substrate to produce eugenol catalyzed by a specific enzyme EGS [37]. Elevated transcript levels of *PmBAHD3* and *PmEGS1* from BS to WS also showed a strong correlation with the emission level of eugenol. Due to its higher expression, *PmEGS1* might be responsible for eugenol synthesis in mei. By integrating the transcriptome database with the emission levels of volatile compounds, a potential biosynthetic pathway for eugenol in mei was summarized. The expression of *PmBAHD5* and *PmBAHD3* played important roles in the synthesis of benzyl acetate from benzyl alcohol, displaying an overall uptrend from BS to WS (Fig. 4B). Interestingly, PmBAHD3 played a role in the synthesis of both benzyl acetate and eugenol because of the substrate diversity of acyltransferases. Whether other members of the alcohol acyltransferase gene family in this study, such as *PmBAHD2* and *PmBAHD17* also participated in benzyl acetate and coniferyl acetate biosynthesis still needs to be further explored. In addition, the release of floral aroma volatiles might be influenced by cellular structure of the petals from BS to FS. More cell–cell spaces were formed in mei petals at FS than those at BS due to a significant increase in proportion of PCs exhibiting a lax arrangement (Fig. S5a, see online supplementary material). This structure might make the emission of fragrant compounds easier in mei petals.

Genes involved in the pathway of a given floral organic volatile compound might be expressed in the same scent-producing cells. This study explored the expression sites of these relevant genes at the cellular level using single-cell expression profiles in mei petals. The process by which PmPAL catalyzes the conversion of phenylalanine to *trans*-cinnamic acid may occur in all petal cell types because of *PmPAL2* nonspecific expression. *Trans*-cinnamic acid was used as a substrate to produce eugenol through several enzymatic reactions catalyzed by enzymes encoded by *PmC4H*, *Pm4CL1*, *PmCCR1*,*2*,*3*, and *PmCAD1*, *PmBAHD3*, and *PmEGS1* genes, exhibiting similar expression patterns as *PmPAL2*, indicating that these successive catalytic actions might occur in all cell types in petals, except for SECC, suggesting that the entire set of genes involved in eugenol production might play synergistic roles in a single cell of every cell type in petals and do not exhibit cell type specificity. Among these genes, *PmCAD1* and *PmBAHD3* showed high expression in XVFs than in others at FS, probably because eugenol formation shares the same initial biosynthetic steps with the lignin biosynthetic pathway [6]. PmBAHD3 and PmBAHD5 converted benzyl alcohol to benzyl acetate in *P. mume* [7], and their encoded genes were also expressed in all cell types, except for SECC, indicating that benzyl acetate might be synthesized in ECs, PCs, and most vascular bundle cells. Interestingly, *PmBAHD5* expression was higher in xylem cells than in other cell types. It was speculated that benzyl acetate synthesized in xylem cells would be transported from petals to stamens for release. This could explain why stamens are the main site for the release and storage of benzyl acetate in addition to petals [38], although *PmBAHD5* expression is much higher in petals than in stamens [7], indicating that the expression site of genes in BP pathway might be related to the release of volatile compounds. Moreover, it was speculated that the expression of genes involved in the major Phe-derived floral volatile biosynthesis mainly occurs at different sites in plants. *NaPAL4* and *Na4CL1* from *N. attenuata* in benzyl acetone (C_6_-C_4_) biosynthesis derived from Phe are highly expressed in ECs and PCs but not in vascular tissues [13], which are not the same as those in mei petals.

In conclusion, we constructed the first single-cell gene expression atlas of woody plant petals using scRNA-seq and established the marker genes for each cell type in mei petals. Moreover, our results indicated that the floral volatile synthesis of mei may occur in ECs, PCs, and most vascular tissue cells in petals. These findings can provide references for further exploring cell types in woody plant petals and understanding the potential molecular mechanism of floral volatile synthesis. However, this study still faced some challenges. For example, it lacked well-established laser microdissection procedures to isolate different cell types in petals, limiting the study at the single-cell level. It was also difficult to obtain the precise contents of various compounds in different cell types. This is critical for a comprehensive understanding of floral volatile biosynthesis.

## Materials and methods

### Plant growth conditions and petal protoplast isolation

‘FHZS’ petals were grown on the campus of Northwest A&F University. Flowers at BS and FS were harvested. Twenty petals were cut into strips before immersion in a 10 mL enzyme solution [2% cellulase R10, 2% macerozyme R10, 0.4 M mannitol, 20 mM KCl, 20 mM MES (pH 5.7), 10 mM CaCl_2_, 0.1% bovine serum albumin, and 0.5 mM dithiothreitol]. Protoplasts were released after shaking at 60 rpm for 2 h at 25°C in the dark at room temperature. The enzyme containing protoplasts was diluted with the same amount of W5 solution [2 mM MES (pH 5.7), 154 mM NaCl, 125 mM CaCl_2_, and 5 mM KCl], and the reaction was terminated. The protoplast solution was filtered by wetting 40 μm nylon with W5 solution and centrifuged at 100 × *g* at 4°C for 6 min. The protoplasts were gently washed twice with W5 solution to obtain pure protoplasts. Cell density and viability were determined with 0.4% trypan blue staining, and protoplasts with a viability of ≥90% and a concentration of ≥1000/μL were immediately processed with the 10X Genomics Single Cell Protocol.

### scRNA-seq library construction, sequencing, and raw data quality control

The library was constructed using the Chromium controller and Chromium Next GEM Single Cell 3'eagent Kits version 3.1. The cell suspension was loaded on a 10X Genomics GemCode single-cell instrument to generate single-cell GEMs (gel beads in emulsion). The scRNA-seq library was prepared using Chromium single-cell 30 gel beads and library kits. The library was pair-ended sequenced on the Illumina sequencing platform. In the scRNA-seq dataset, Read1 contained a 16 bp GemCode barcode and 10 bp UMI, and Read2 was a cDNA sequence fragment.

Using 10X Genomics Cell Ranger (version 3.1.0), reads with low-quality barcodes and UMI were filtered out, FASTQ sequencing reads converted from raw data were mapped to the *P. mume* var. *tortuosa* genome, and a digital gene expression matrix was generated. The gene-cell matrix was loaded into the Seurat package (version 3.1.1) for further quality control, and cells with UMIs ≥8000 or mitochondrial gene percentage ≥10% and expressed <500 or >4000 genes were filtered out. Bimodal cells were removed by DoubletFinder (version 2.0.3). After removing low-quality cells, expression was further homogenized and Harmony was used to merge data to generate the original data for further analysis. R was used to calculate the Pearson correlation coefficient between bulk RNA-seq and scRNA-seq datasets.

### Statistics for differential gene expression analysis

Seurat was used to perform differential gene expression analysis of genes in different cell types of ‘FHZS’ petals at BS and FS. The screening standards were as follows: |log_2_FC| ≥0.36, the proportion of cells expressing target genes in each group ≥0.1, and an adjusted *P* value (*P*_adj_) ≤0.05. The genes were tested for significance using MAST, and *P* values were adjusted by the Benjamini–Hochberg method.

### Bulk RNA-seq library construction and sequencing

Total RNA was extracted from petals using Trizol reagent according to the manufacturer’s instructions. Each sample contained three replicates. High-quality RNA samples with RIN number >7.0 were screened out by Bioanalyzer 2100 and RNA 6000 Nano Lab Chip Kit (Agilent, Santa Clara, CA, USA) for RNA-seq library construction. Sequencing was conducted by an Illumina Novaseq™ 6000. Clean reads were aligned to the mei reference genome using HISAT2 [39], and expression of all transcripts was estimated by StringTie and ballgown [40].

### Collection and determination of floral volatiles in *P. mume*

‘FHZS’ petals at four flowering development stages were collected. After being weighed, petals from different flowering development stages were separately placed into 25 mL injection vials and then held for 10 min before using extraction fiber to absorb the volatiles for 30 min at 30°C. GC–MS was used to analyse the emission amounts of floral volatiles. Helium was the carrier gas in the split mode with a split ratio of 20 and a column flow rate of 27.0 mL/min. The temperature of the GC oven started at 40°C, was maintained for 2 min, increased to 250°C (at a speed of 5°C/min), and held for 6 min.

### *In situ* hybridization

‘FHZS’ petals at BS and FS were collected and immediately put into an FAA solution (50% ethanol, 5% acetic acid, and 3.7% formaldehyde) for fixation for >12 h. The antisense probe of genes was generated with gDNA as a template according to the DIG High Prime Lab/Detection K (Roche). The primers are shown in Table S7 (see online supplementary material). *In situ* hybridization was performed as described previously [41]. Images were observed using a Leica MZ10F microscope (Leica Co., Baden-Württemberg, Germany).

### Heterologous expression of PmBAHD3 protein in *E. coli* and *in vitro* enzymatic assay

To express the PmBAHD3 protein in *E. coli*, the full-length sequence of the *PmBAHD3* gene was amplified with gene-specific primers and seamlessly cloned into the *pCold-TF* vector. The recombinant plasmid *pCold-TF-PmBAHD3* and the empty *pCold-TF* vector were separately transformed into *E. coli ArcticExpress* (DE3). After induction with 0.5 mM isopropyl-β-D-thiogalactopyranoside at 12°C for 20 h, cells were harvested by centrifugation, resuspended in phosphate-buffered saline (pH 7.2), and broken down by sonication. The recombinant pCold-TF-PmBAHD3 protein and pCold-TF protein were purified using a nickel-Sepharose column (MedChemExpress, USA). The purified products were analysed and confirmed by sodium dodecyl sulfate-polyacrylamide gel electrophoresis and used for subsequent enzyme activity analysis.

Enzyme activity was determined in a 200 μL reaction mixture by mixing 50 μg purified PmBAHD3 protein with 1 mM benzyl alcohol, 250 μM acetyl-CoA, and 10 mM MgCl_2_ in 50 mM Tris–HCl (pH 8.0). The mixture was incubated at 30°C for 1 h, followed by the addition of 100 μL ethyl acetate to the reaction under vortexing and centrifugation. The supernatant was transferred to a sample vial for GC–MS analysis.

## Supplementary Material

Web_Material_uhae189
