# Supplementary material for: Single-cell RNA sequencing reveals a high-resolution cell atlas of petals in Prunus mume at different flowering development stages
Source: Hortic Res. 2024 Jul 10;11(9):uhae189. doi: 10.1093/hr/uhae189 (PMC11377181; doi:10.1093/hr/uhae189)
Supplement: Web_Material_uhae189 [file web_material_uhae189.zip › Supplementary Figures-revised.docx]

**Single-cell RNA sequencing reveals a high-resolution cell atlas of petals in *Prunus mume* at different flowering development stages**

Yuhong Guo^1, #^, Xiling Chen^1, #^, Jinhong Li^1^, Qi Wang^1^, Shuangyu Zhang^1^, Nuoxuan Liu^1^, Yanlong Zhang^1^ and Tengxun Zhang^1,*^

^1^ College of Landscape Architecture and Arts, Northwest A&F University, Yangling, China

**^*^Correspondence:**

Tengxun Zhang, ztxnwafu@nwafu.edu.cn

**^#^** The authors contribute equally.


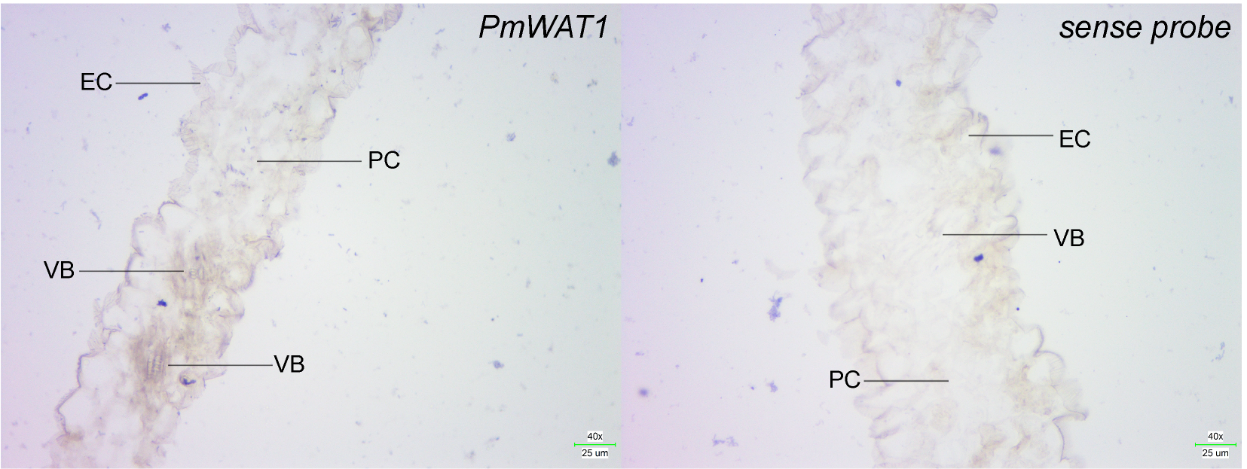
.

**Figure S1.** Localization of *PmWAT1* in ‘FHZS’ petals by *in situ* hybridization. Bar=25μm. EC, epidermal cell; PC, parenchyma cell; VB, vascular bundles.


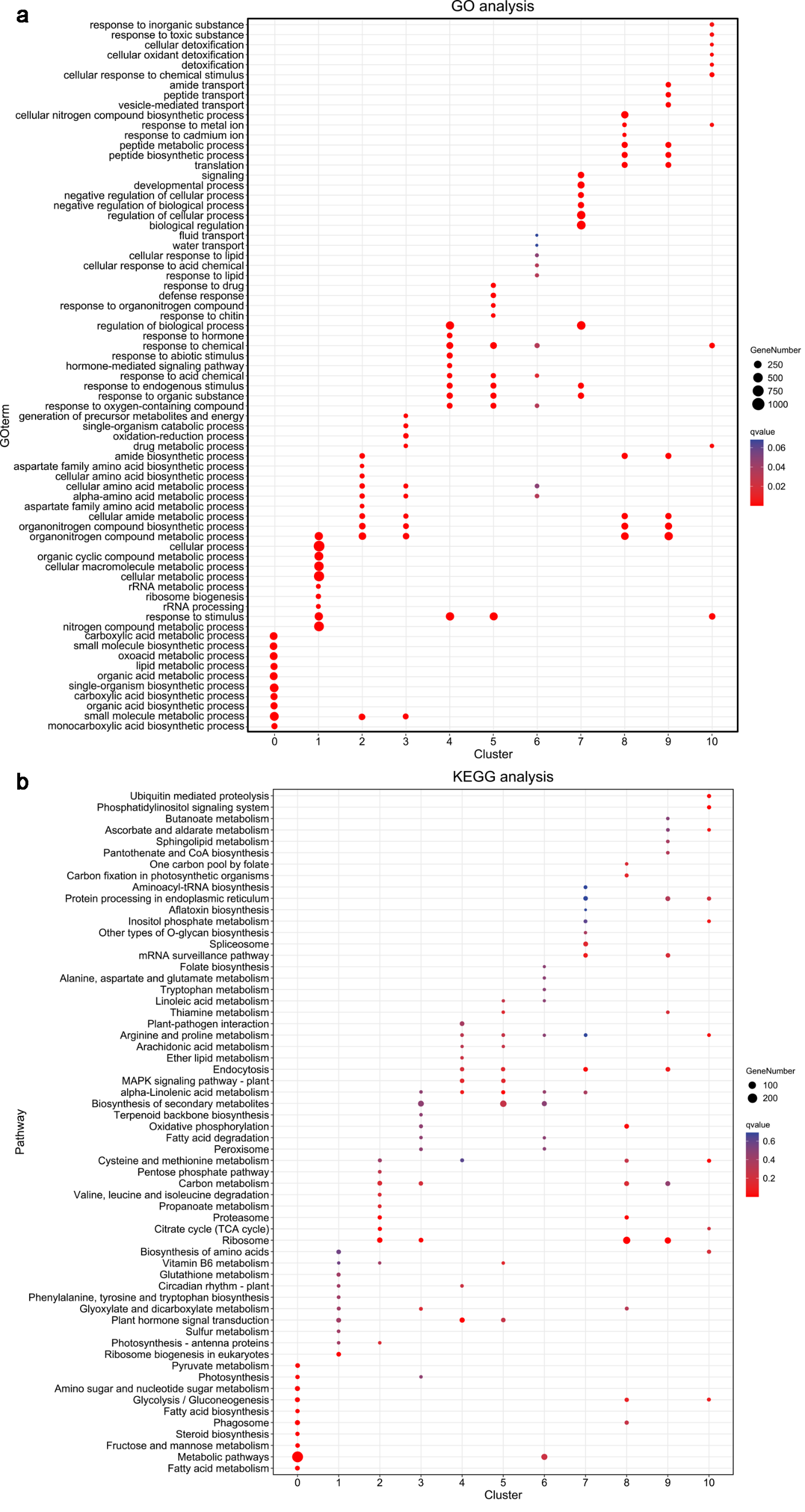


**Figure S2.** These cell type-specific DEGs in 11 clusters were enriched in various biological processes in GO terms (a) and KEGG annotation (b), p ≤ 0.05. Dot size represent the number of genes enriched in the corresponding pathway and the color represent q-value change.


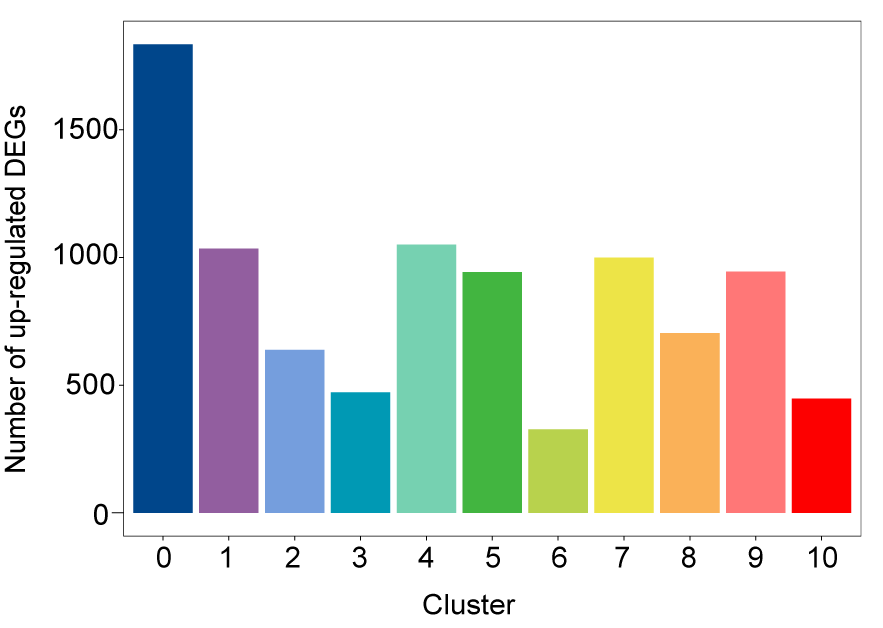


**Figure S3.** The number of relevant up-regulated DEGs in different clusters. The genes must be expressed in 25% of cells within the target cluster; q ≤ 0.01; log_2_FC ≥ 0.36. Epidermal cells (0); Parenchyma cells (1, 2); Xylem parenchyma cells (3, 5); Phloem parenchyma cells (4); Xylem vessels and fibers (8); Sieve elements and companion cells complex (10); Unknown cells (6, 7, 9).

**
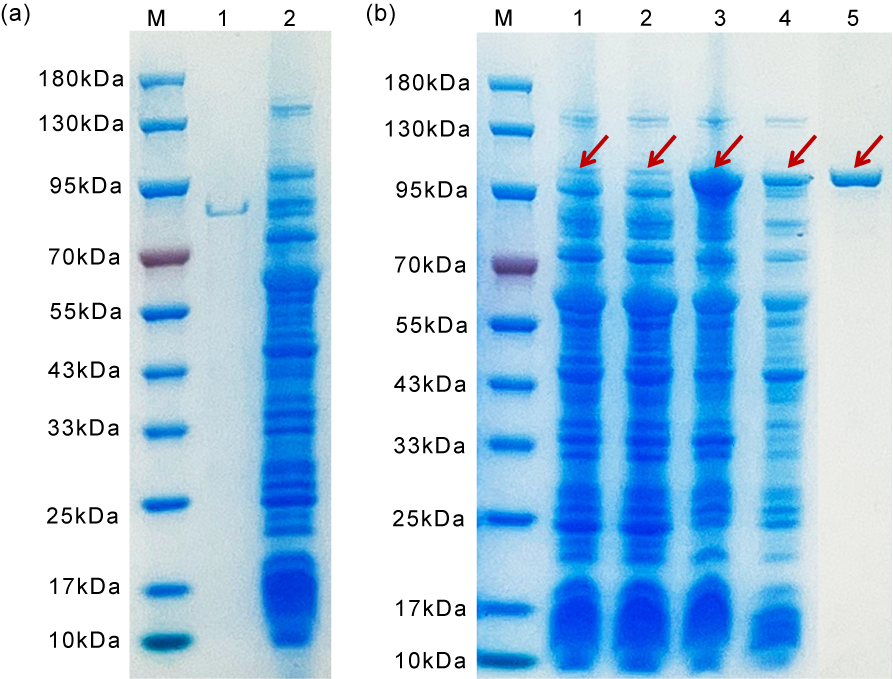
**

**Figure S4.** SDS-PAGE analysis of recombinant PmBAHD3. (a) SDS-PAGE analysis of pCold TF: Lane M: protein marker; Lane 1: fractions eluted by imidazole solutions with 250mM concentrations; Lane 2: uninduced *E. coli* *ArcticExpress* (DE3) containing pCold TF. (b) Visualization of the expression and purification of PmBAHD3 protein on 12% SDS-PAGE. Lane M: protein marker; Lane 1: uninduced *E. coli* *ArcticExpress* (DE3) containing pCold TF-PmBAHD3; Lane 2: induced *E. coli ArcticExpress* (DE3) containing pCold TF-PmBAHD3; Lane 3: insoluble fraction (pellet); Lane 4: soluble fraction (supernatant), Lane 5: purified protein of PmBAHD3.


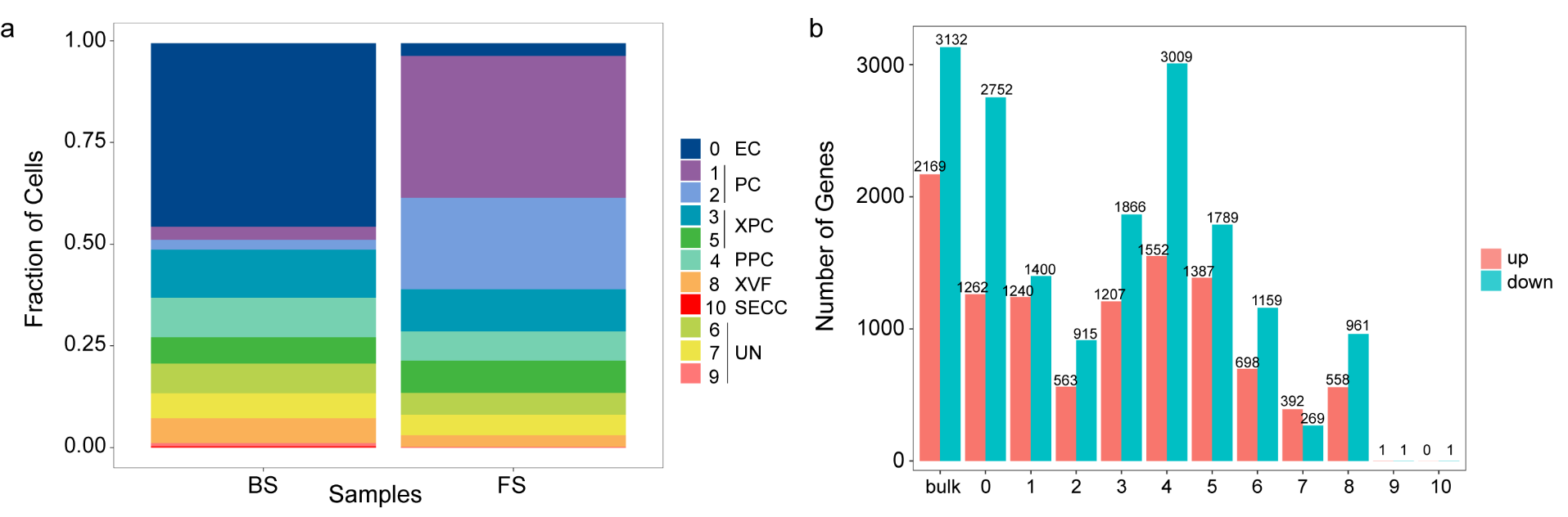


**Figure S5.** The percentages of cell types (a) and the number of differential expression genes in different cell types of mei petals (b) at budding and full-blooming stages. BS, budding stage; FS, full-blooming stage. Bulk, all cells obtained; Epidermal cells (0); Parenchyma cells (1, 2); Xylem parenchyma cells (3, 5); Phloem parenchyma cells (4); Xylem vessels and fibers (8); Sieve elements and companion cells complex (10); Unknown cells (6, 7, 9); The differentially expressed genes were selected with |log2FC| ≥ 0.36, the proportion of cells expressing target genes in each group ≥ 0.1, and an adjusted p value ≤ 0.05.

**
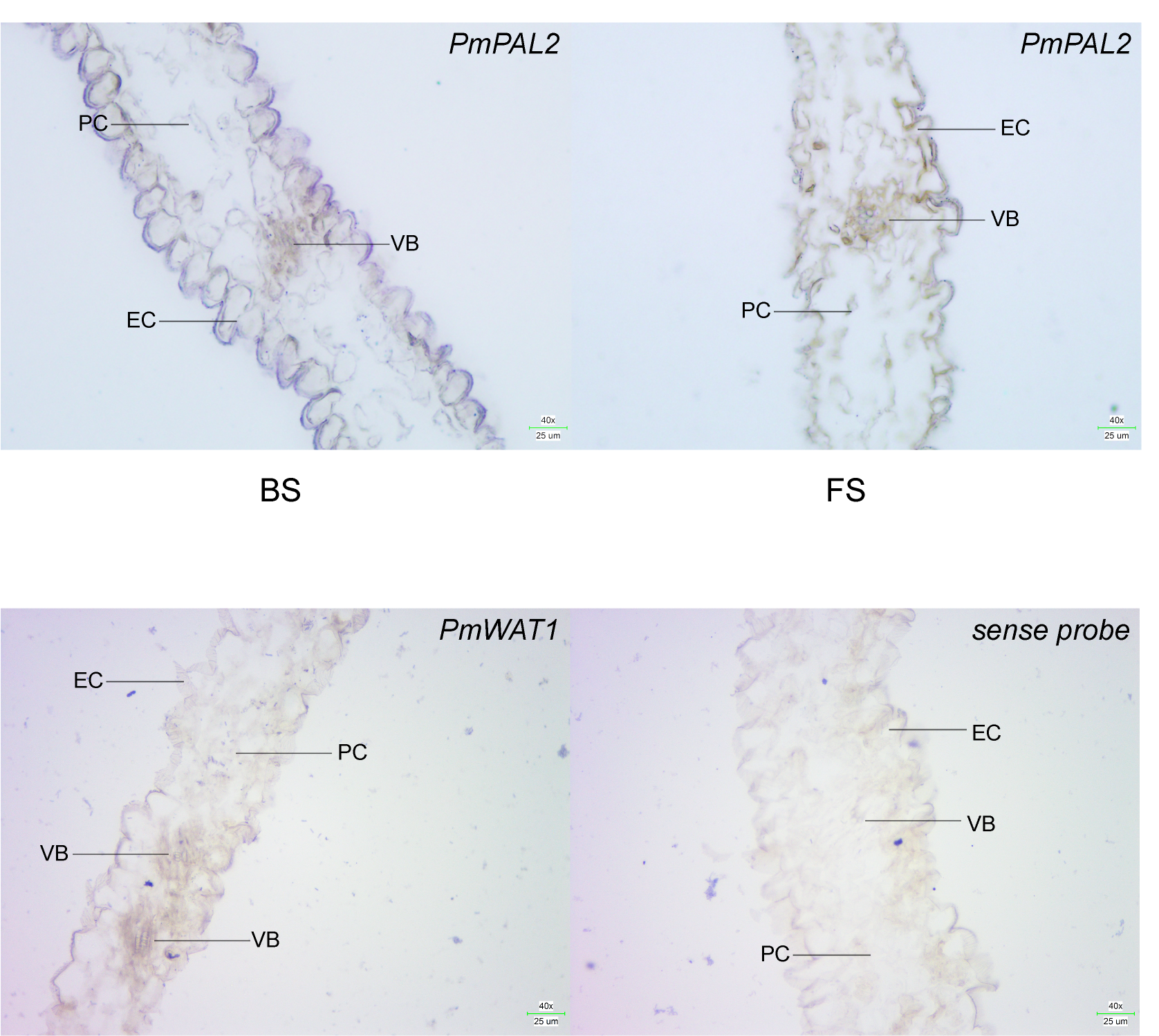
**

**Figure S6.** Localization of *PmPAL2* in ‘FHZS’ petals by *in situ* hybridization at budding and full-blooming stages. Bar=25μm. BS, budding stage; FS, full-blooming stage; EC, epidermal cell; PC, parenchyma cell; VB, vascular bundles.

**
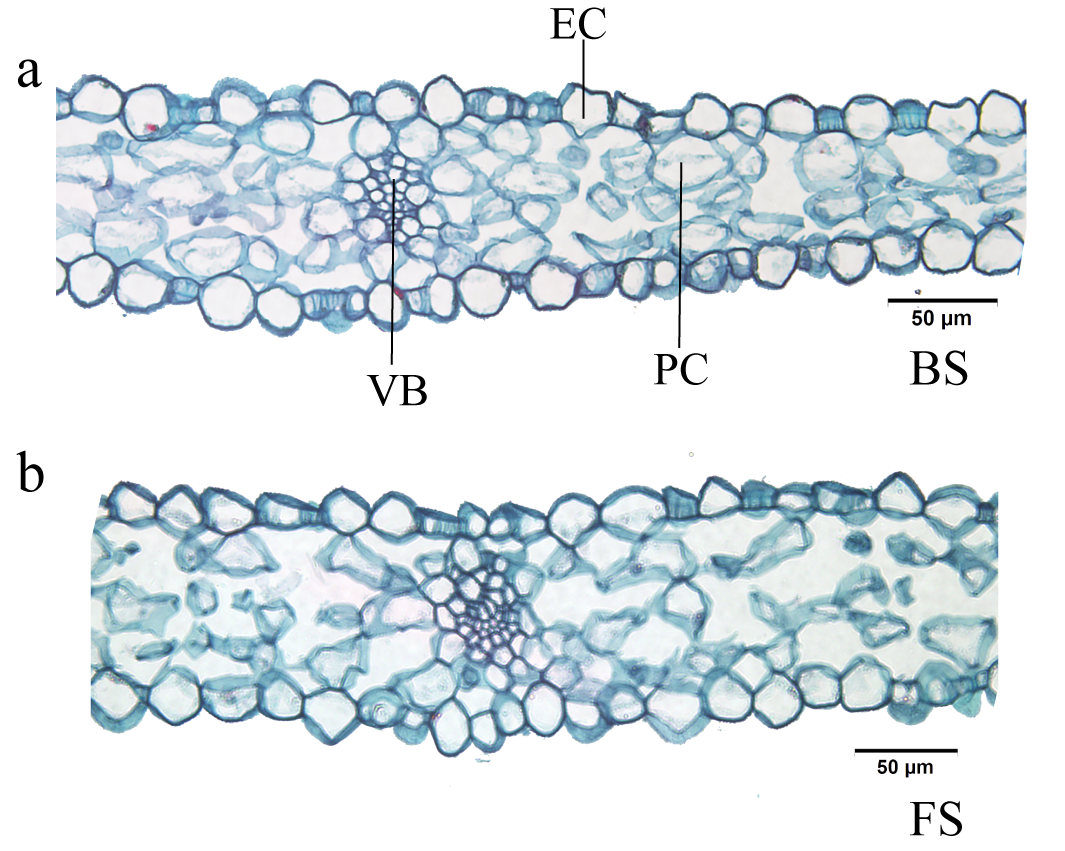
**

**Figure S7.** The stained sections of ‘FHZS’ petals under the microscope with visible light at budding stage (a) and full-blooming stage (b), respectively. Bar=50μm. BS, budding stage; FS, full-blooming stage; EC, epidermal cell; PC, parenchyma cell; VB, vascular bundles.
